# The Childhood Autism Rating Scale Second Edition (CARS2) and Its Applicability in an Iranian Sample

**DOI:** 10.1002/aur.3309

**Published:** 2025-02-03

**Authors:** Sayyed Ali Samadi, Ameneh Mahmoodizadeh, Mehdi Foladgar, Shahnaz Bakhshalizadeh Moradi, Baran Lotfi, Roy McConkey

**Affiliations:** ^1^ Institute of Nursing and Health Research Ulster University Belfast UK; ^2^ Iranian Special Education Organization Iran; ^3^ Ordibehesht Autism Education and Rehabilitation Center Isfahan Iran; ^4^ Raha Autism Education and Rehabilitation Center Tabriz Iran; ^5^ Baran‐e Mehr Autism Education and Rehabilitation Center Neyshabur Razavi Khorasan Iran

**Keywords:** autism spectrum, childhood autism rating, cultural adaptations, Iran, Psychometrics, scale second edition (CARS 2), validation

## Abstract

The present study aimed to evaluate the reliability and validity of the Childhood Autism Rating Scale: Second Edition (CARS2) in diagnosing individuals with autism in Iran. A mixed‐method approach was used and 313 participants were recruited, with an age range of 2–32 years, for CARS2‐Standard Form (ST) and 218 individuals aged 6–25 years for CARS2‐High Functioning (HF). The participants were recruited from daycare centers, schools, and clinics with different developmental trajectories: autism, intellectual disabilities, and neurotypical development. All participants with autism and intellectual disabilities had been clinically diagnosed previously. In addition, the CARS2‐Questionnaire of Parent Concerns (QPC) was used to gather qualitative data on 30 randomly selected parents and the perspectives of the 20 test administrators were also collected. The CARS2 had high internal consistency, inter‐rater reliability, and test–retest reliability. Factor analyses revealed a one‐factor structure for CARS2‐ST and a three‐factor structure for CARS2‐HF. When adjustments were made to the cut‐off points, the discriminant analyses indicated that CARS2 effectively differentiated between those with autism and typical development but less so with persons who had intellectual disabilities. The qualitative data analysis and the extracted themes suggest that the CARS2‐QPC is a valid tool for collecting autism‐related information from parents. Our findings suggest that the CARS2 is broadly a reliable and valid instrument for diagnosing autism spectrum in Iran in the absence of more extensive assessments.

**Summary**

- Researchers in Iran used a tool called CARS2 to see whether it helps to identify children and adults with Autism.
- They tested CARS2 on over 500 individuals, aged 2–32.
- A group of the population had autism, some had intellectual disabilities, and some were developing typically.
- The study showed CARS2 is an applicable tool because different testers got similar results and these were consistent over time (it was a reliable tool).
- It helped identify the difference between the three groups (it gave valid results).
- Parents also found CARS2 helpful for sharing information about their children.
- Overall, CARS2 with some changes would be a useful tool for diagnosing autism in its different forms in Iran.

## 1 Introduction

Autism is characterized by impairments in social interaction, communication, and repetitive patterns of behavior (APA 2022). It affects early development (Goodwin, Matthews, and Smith 2019; Hodges, Fealko, and Soares 2020) and is considered a lifelong neurodevelopmental disorder (Charman 2014). Autism affects individuals worldwide, from different socioeconomic backgrounds and ethnicities and that is also evident in Iran (Salari et al. 2022).

The prevalence of autism internationally has increased in recent years but with wide variations across countries (Zeidan et al. 2022). In the United States, a very high prevalence rate of 1 out of 36 children with autism has been reported (Harris 2023), whereas rates are much lower in many low and middle‐income countries. These discrepancies have been attributed to variations in knowledge about autism in the population, the lack of professionals to undertake diagnosis and treatment, and the absence of suitable assessment tools (Durkin et al. 2015). Yet, the assessment and diagnosis of autism is crucial to providing appropriate interventions and support for individuals with this condition (Koegel et al. 2014).

The diagnosis process remains a challenge in many low‐ and middle‐income countries, especially as scales developed in English‐speaking, western cultures are implemented without considering their validity, cultural sensitivity, and the provision of adequate training of the assessors (Gau et al. 2013; Pacífico et al. 2019; Samadi, McConkey, and Mahmoodizadeh 2021). One widely used assessment tool has been the Childhood Autism Rating Scale (CARS) (Schopler, Reichler, and Renner 1988), and the publication of the second edition (Schopler et al. 2010) has also started to be used in various countries with promising results (Alotaibi and Alotaibi 2021; Stevanovic et al. 2021). A wealth of evidence suggests that the CARS provides reliable and valid assessments of ASD symptomatology in both research and clinical settings worldwide, including regions with varying income levels (e.g., Breidbord and Croudace 2013; García‐Lopez and Narbona 2014; Mayes et al. 2014; Moulton et al. 2019; Park and Kim 2016; Russell et al. 2010; Samms‐Vaughan et al. 2017; Santos et al. 2012). The reviews undertaken by Moon et al. (2019) and Randall et al. (2018) of these and other studies confirmed the sound psychometric properties of the CARS and have identified moderate levels of specificity as a standalone ASD diagnostic tool. Due to the scale's good psychometric properties, ease of implementation in various settings, its cost‐effectiveness, and the manageable training requirements, the CARS has been considered as a suitable and sustainable evaluation/diagnostic tool to address socioeconomic and geographic gaps in autism identification, particularly in low‐resource regions (e.g., Samms‐Vaughan et al. 2017). However, uncertainty remains regarding the applicability of the updated version of the scale and the extent to which autism is defined and measured consistently across different societies and world regions due to a lack of data on its cross‐cultural use.

This study aimed to explore the applicability of the Childhood Autism Rating Scale, 2nd version, in assessing individuals with autism in Iran. This is essential for several reasons. Firstly, it will enable professionals in Iran to more accurately identify and assess individuals with autism, which should lead to more targeted interventions and support services. Secondly, it will provide further insights into the way autism may be perceived differently in Iranian cultures and the need to develop culturally sensitive interventions and educational programs. Thirdly, the processes used to validate CAR2 for use in Iran would provide a model that other cultures could follow in assessing the validity of this and other assessment tools for use in their countries.

Therefore, the main aim of the study was to evaluate the applicability of the CARS2 in assessing individuals with autism within the Iranian context in order to enhance autism identification and to facilitate targeted interventions and support services. Our wider objectives included the training needs of assessors who use the scale; exploring the cultural nuances in autism perception within the Iranian population so as to inform the development of culturally sensitive interventions and educational programs; and establishing a model for validating assessment tools in different cultural settings.

To achieve these objectives, this study employed a mixed‐methods approach, combining quantitative data from the administration of the CARS2 with qualitative data gathered through interviews with parents using CARS2‐QPC, and focus groups with parents, educators, and clinicians working with individuals with autism in Iran.

## 2 Method

### 2.1 Childhood Autism Rating Scale 2nd Edition (CARS2)

The updated version of CARS2 divides the original CARS scale into two versions. One version (CARS2‐HF) is intended to identify individuals with high‐functioning autism, those with a fluency in verbal ability from 6 years onward and with an IQ greater than 80. The diagnosis of individuals with high‐functioning autism has been a particular challenge in Iran due to the shortage of valid diagnostic instruments. Hence, this study will have significant clinical implications in Iran and possibly neighboring countries.

The second version is labeled the standard version (CARS2‐ST) and is intended for use with children younger than 6 years and older individuals who have less than average IQ and/or notable communication difficulties. The original CARS and CARS2 have consistently shown good diagnostic agreement with the DSM‐III‐R (Nordin, Gillberg, and Nyde'n 1998), DSM‐IV (Perry et al. 2005), and DSM‐5 in North American samples (Dawkins, Meyer, and Van Bourgondien 2016).

CARS2 also includes an unscored section called the Questionnaire for Parents or Caregivers (CARS2‐QPC) that collects information from those who know the child well. Their ratings need to be integrated with other information gathered by the assessors through individual interviews with the primary caregiver and direct observation of children when making their ratings on the CARS2‐ST and ‐HF items.

The two versions of CARS2 each consist of 15 items that enable the rater to score the individual based on a Likert system that starts from 1 (within normal limits for that age) through 2, 3 to 4 (severely abnormal for that age) but also with three midpoints (1.5, 2.5 and 3.5) that indicates an item that falls between two categories.

The scores of each item are summated to obtain a total score, which ranges from 15 ti 60. Based on the standardization sample of children in the USA, a diagnosis of autism is made based on the total scores: for CARS2‐ST, a score of > 30 for under 13 years individuals and > 28 for over 13 years old indicates a high probability of autism. For the CARS2‐HF, a total score of > 27.5 indicates a high probability of autism (Schopler et al. 2010; Geier, Kern, and Geier 2013).

### 2.2 Other Scales Used

The data from three other assessment tools were obtained from prior records and used alongside the CARS2 in this study to gain insights into its validity

- *The Wechsler Intelligence Scale for Children‐ Fourth Edition*. For individuals aged 6–16 years old, the Iranian version of the Wechsler Intelligence Scale for Children‐ Fourth Edition (Shsrifi and Rabiei 2012) was used to identify children over six who were verbally fluent and had a normal range of IQ (estimation of 80 or higher).
- *The Autism Diagnostic Interview‐Revised (ADI‐R*). The Farsi version of the ADI‐R (Samadi, McConkey, and Mahmoodizadeh 2021) is considered a well validated and “gold standard” instrument to assist with a diagnosis of autism.
- *The Gilliam Autism Rating Scale‐ 2*nd *version* (GARS2). The Farsi version of GARS2 (Samadi and McConkey 2014) was used for those children who did not have access to diagnostic scales such as ADI‐R.

## 3 Procedure

The study was conducted under the Declaration of Helsinki. We adhered to the seventh revised version on Medical Research involving human subjects issued on October 19, 2013. Caregivers were provided with a written comprehensive information sheet and a consent form that detailed the purpose of the study, the specific procedures involved, the potential risks and benefits, as well as their right to withdraw from the study at any point without any impact on the services they were receiving. The caregivers demonstrated their consent by completing and signing a demographic questionnaire, which was then submitted to the data collection centers.

### 3.1 CARS2 Translation Process

The first author translated all the items of CARS2‐ST, CARS2‐HF, and CARS2‐QPC, into Farsi. Back‐translations to English were undertaken by an independent bilingual speaker experienced in learning disabilities. A fluent English and Farsi speaker and psychology lecturer then adjusted the Farsi translation to accord better with the original English scale. The modified Farsi version was further finalized after testing it with five parents of children with developmental delays in a clinical setting.

### 3.2 CARS2 Training Course

The assessors for the study were recruited from the autism centers under the supervision of the Iranian Social Welfare Organization (ISWO) and 20 centers volunteered to participate. At each center, one experienced person was identified, with an average of 8.2 years of service in the Center's assessment and evaluation section. Each assessor had already been trained and certified in the evaluation and application of GARS2, ADI‐R, or both, by the Iranian Special Education Organization (ISEO) and ISWO (*n* = 20).

Based on the qualification guideline for training to administer CARS2, it is considered a level C scale which is equates to undergraduate level and for which a certification from an agency/organization is needed. In this study, three (15%) assessors were graduate students in Master's degree programs, nine had Master's degrees (45%), and eight held PhDs (40%). The three graduate students possessed at least 5 years of relevant work experience. Hence no one needed external certification to undertake CARS2 assessments.

However, within this study and to align with its objectives, all the assessors participated in a training course on administration and scoring both versions of CARS2. They participated in four online sessions comprised of different observations and coding of mock scenarios using two videos on both CARS2‐ST and ‐HF as well as ‐QPC being administered with the persons and family carers. The 20 assessors were then required to undertake five assessments of persons using CARS2 over a two‐month period during which they received online supervision, shared videos, and took part in group discussions related to the observation and coding of the CARS2 items.

### 3.3 Assessment Process

The administration process of CARS2 with study participants took 6 months. The assessors recruited persons from their daycare centers under the supervision of ISWO, all of whom had already been officially diagnosed to have autism using ADI‐R or GARS2. The diagnostic data were obtained from the children's clinical records, and for the group who were considered as having high‐functioning autism, their WISC‐IV data or an approved, documented estimate of IQ by the evaluators was also obtained.

Parents of the individuals with autism were notified about the study, and the process was explained to them officially in both written and oral format. Both scales of the CARS2 were administered in the evaluation department of the autism center in which the person attended. Before this appointment, parents were given the CARS2‐QPC scale in a sealed envelope and asked to fill out the form at home and hand it back to the evaluator in a sealed envelope when they brought their relative to the center for the CARS2 assessment.

In addition, the sample of children with intellectual disabilities was recruited from the daycare centers under the supervisor of ISWO in each city. The typically developing sample of children were recruited from the kindergartens and schools in the same city all of whom had no clinical diagnosis based on their registration documents. With these two groups, the same procedures were followed for providing information and consent taking and the administration of CARS2.

To evaluate the level of the test–retest reliability of the two versions of CARS2, 56 individuals with autism were retested on CARS2‐ST after a time interval of three weeks. Their ages ranged from 3 to 28 (mean = 7.44, SD = 3.48). Likewise, 50 individuals assessed using CARS2‐HF were retested. Their ages ranged from 7 to 23 years (mean = 10.16 SD = 4.16).

To assess inter‐rater reliability, 10 evaluators from participating autism centers exchanged to re‐rate persons in a different center. The second raters had access to the same clinical records, interviews, and behavioral observations but not the ratings given by the first assessor. For CARS2‐ST, 46 individuals with autism were re‐assessed (age range of 3–32 years: mean = 7.82 SD = 6.59), and for CARS2‐HF, 45 individuals were re‐assessed (age range of 6–23: mean = 9.54, SD = 3.99).

As CARS2‐QPC is not considered to contribute to the scoring process in determining a diagnosis of autism, no retest and inter‐rater reliability procedures were undertaken on this scale.

The second assessment data of the participants included in the reliability analyses (test–retest and inter‐rater reliability) were not included in the results for the full cohort which used only the scores from the first assessment. In addition to the statistical difficulties this would generate for including two scores for some individuals, it replicated the standard assessment protocols in which reassessment is rarely undertaken.

The entire study, including assessor training, participant recruitment, initial and test–retest assessments, was completed within a six‐month period in 2023/24.

## 4 Results

### 4.1 Quantitative

#### 4.1.1 Sample Characteristics

In all, 531 individuals with different developmental trajectories from different geographical locations across the county were recruited. They consisted of neurotypically developing children and those with a formal diagnosis of two main types of developmental disabilities, namely autism and intellectual disabilities “ID.”

The autism sample in total consisted of 344 individuals. The contrast samples comprised 187 persons, 97 individuals with a clinical diagnosis of intellectual disability (ID) and 90 children as the “neurotypical developing” and no clinical diagnosis. For the CARS2‐HF comparison, only neurotypical developing children and those with autism who were aged 6 years and above and IQ≥ 80 were recruited. However, for the CARS2‐ST neurotypically developing participants along with ID individuals were considered as the control groups.

#### 4.1.2 CARS2‐ST Version

This sample consisted of 313 individuals with an age range of 2–32 years (Mean = 7.71, SD = 4.24) in three different developmental groups of individuals, those with autism (*n* = 166, 53%), intellectual disabilities (*n* = 97, 31%), and typically developing (*n* = 50, 16%). They were recruited from different services in 30 cities located in 17 provinces across the country.

#### 4.1.3 CARS2‐Hf

In all, 218 individuals aged from 6 to 25 years (Mean = 10.64, SD = 3.82) in two developmental groups were recruited from 22 cities located in 14 provinces. The sample consisted of 178 (82%) individuals with autism and 40 (18%) neurotypically developing individuals.

All participants recruited for the study were from urban areas. For those assessed with CARS2‐ST (*n* = 313), 258 (82%) were from the Farsi speaking provinces and 55(18%) from Non‐Farsi speaking provinces. For the CARS2‐HF group (*N* = 218), 182 (83%) were from the Farsi speaking provinces 36 (17%) from Non‐Farsi speaking provinces. While cultural and linguistic variations exist within Iran, the use of Farsi as the assessment language ensured consistency across all participants.

The demographic information of these samples is presented in Table 1.

**TABLE 1:** The demographic information of the participant samples for the CARS2‐ST and HF versions.

Version	Number and percentages	Age Mean and SD	Sex
CARS2‐ST	Autism (*n* = 166, 53%) Intellectual Disabilities (*n* = 97, 31%) Neurotypically developing (*n* = 50,16%)	7.28 (3.77) 8.39 (5.14) 7.82 (3.87)	Male (*n* = 122,73.5%) Female (*n* = 44,26.5%) Male (*n* = 73,75%) Female (*n* = 24,25%) Male (*n* = 28,56%) Female (*n* = 22,44%)
CARS2‐HF	Autism (*n* = 178, 82%) Neurotypically developing (*n* = 40, 18%)	10.44 (4.08) 11.55 (2.15)	Male (*n* = 147, 83%) Female (*n* = 31, 17%) Male (*n* = 26,65%) Female (*n* = 14,35%)

### 4.2 Data Analysis

SPSS 25 was used for statistical analyses, starting with the internal consistency of the CARS2 scales, test–retest reliability, and inter‐rater reliability. Cronbach's *α* was used to determine the Internal consistency of the scales. The test–retest reliability and inter‐rater reliability were examined through intraclass correlation coefficients (ICC) for each of the 15 items using a two‐way random effects model suggested by Shrout and Fleiss (1979) based on the total scores reported in this study. Koo and Li (2016) proposed that “poor” Alpha values were less than 0.5, “moderate” were values between 0.5 and 0.75, “good” values between 0.75 and 0.9, and “excellent” reliability, if the values were greater than (0.90).

Factor analyses were conducted with the autism groups' data to confirm the construct validity of the two CARS2 versions with Iranian samples. A principal component analysis was conducted to extract the factors across the 15 test items, and the eigenvalues and explanatory variance ratios were examined for each factor. To maximize the sum of the variance of the squared loadings, a varimax rotation with Kaiser normalization was also used.

The concurrent validity of the CARS was examined by calculating the correspondence between the autism ratings from the CARS2 with those previously assessed using ADI‐R or from GARS2.

To determine the discriminant validity of the CARS2‐ST and ‐HF, discriminant analyses were conducted for the neurotypically developing group and the autism group. Similar analyses were used examine differences between those with autism and those with intellectual disabilities and between the latter and those who were typically developing. Additionally, the sensitivity and specificity values of the CARS2 classifications were assessed using ROC analyses.

## 5 Results

### 5.1 Reliability

Internal consistency: Cronbach's α coefficient for the total CARS2‐ST was 0.932 and for the CARS2‐HF was 0.907.

Test–retest reliability: A sample consisting of 59 individuals evaluated with CARS2‐ST and 50 individuals evaluated in the CARS2‐HF group were re‐evaluated after 3 weeks by the same assessors and the Pearson product moments correlations between the ratings given on each item were calculated. For the CARS2‐ST, the correlation between the total scores on the 15 items was 0.994 with ICC values for each item ranging from 0.96 to 0.99, with the total score ICC of 0.98.

For CARS2‐HF, the correlation between the total scores on the 15 items was 0.996 with ICC values for each item ranging between 0.93 and 0.99, with the total score ICC of 0.95.

Inter‐rater reliability: The level of agreement between the two independent raters was calculated by correlating the total scores and also the scores for each item given by each assessor to 46 participants in the CARS2‐ST group and 45 participants in the CARS2‐HF. The Pearson's correlation coefficients between total scores were 0.991 for CARS2‐ST and 0.901 for CARS2‐HT with ICC values of each item showing good to excellent reliability between 0.86 and 0.98 for CARS2‐ST and 0.85 and 0.98 for CARS2‐HF. The Kappa result, a measure of inter‐rater agreement beyond chance, for CARS2‐ST was in a range from 0.765 to 0.983 for the 15 items which mean good to excellent and for the CARS2‐HF it was from 0.676 to 988 in a range from moderate to excellent.

As noted below, cut‐off scores are used in CARS2 to identify persons with a high probability of autism. At the categorical cut‐off level, there was near perfect agreement between the two evaluators' judgments for CARS2‐ST: *κ* = 0.936 (95% CI, 0.803–1.062), *p* < 0.001. For the CARS2‐HF, at the categorical level there was less but still good agreement reported between the two evaluators' judgments: *κ* = 0.814 (95% CI, 0.611–1.017), *p* < 0.001.

### 5.2 Validity

Concurrent validity: Correlations between the total scores on the CARS items and those from the ADI‐R or GARS2 were used to indicate the concurrent validity of CARS. For CARS2‐ST, the correlations with ADI‐R total score were *r* = 0.909 (*n* = 71) and with GARS2 was *r* = 0.897 (*n* = 77). Both were highly significant (*p* < 0.001).

For CARS2‐HF, the correlations with ADI‐R total score were *r* = 0.921 (*n* = 37) which was highly significant (*p* < 0.001) but the correlation with GARS total scores was not significant (*r* = 0.02; *n* = 87). This is not surprising as GARS is designed more for use with more severely impaired individuals but it is reported here for the sake of completeness.

Construct Validity: To explore the construct validity of the CARS2, a factor analysis was undertaken of the 15 items on each version of the individuals who had a diagnosis of autism using a principal components analysis and a varimax rotation. Table 2 shows the factor loadings of the items on the CARS2‐ST which together accounted for 55% of the variance.

**TABLE 2:** The factor loading of the CARS2‐ST items (*N* = 313).

Item (number)	1	2	3
Imitation 2	0.800		
Verbal communication 11	0.757		
General impressions 15	0.753		
Object use 5	0.746		
Relating to people 1	0.709		
Emotional response 3	0.679		
Nonverbal communication 12	0.622		
Body use 4	0.527		
Adaptation to change 6		0.715	
Fear or nervousness 10		0.688	
Activity level 13		0.633	
Level and consistency of intellectual response 14		0.583	
Taste, smell, and touch response and use 9		0.409	
Listening response 8			0.768
Visual response 7			0.744
Explanation variance	38.82	10.85	7.86
Cumulative total variance	38.82	49.67	57.53

For this Iranian sample, Factor 1 was the dominant factor and broadly reflected the three main domains in autism of social interactions and communication in which some stereotypical behavioral items also featured. Two other minor factors consisted of more emotional responses and on the person's responsiveness.

A similar analysis was undertaken for the CARS2‐HF items, and a three‐factor solution was identified that accounted for 62% of the variance that aligns with the original conceptualization of the measure, with high loadings on the primary and secondary factors and minimal loadings on the third factor (see Table 3).

**TABLE 3:** The factor loading of the CARS2‐HF items (*N* = 218).

Item (number)	1	2	3
Thinking/cognitive integration skills 13	0.810		
Nonverbal communication 12	0.807		
Verbal communication 11	0.766		
General impressions 15	0.754		
Visual response 7	0.488	0.459	
Emotional expression and regulation of emotions 2		0.749	
Social emotional understanding 1		0.674	
Taste, smell, and touch response and use 9		0.639	
Adaptation to change/restricted interests 6		0.637	0.564
Relating to People 3	0.446	0.568	
Body use 4		0.554	
Object use in play 5	0.404	0.546	
Listening response 8		0.446	
Level and consistency of intellectual response 14	0.521		0.703
Fear or Anxiety 10			0.699
Explanation Variance	27.49	24.39	18.94
Cumulative Total variance	27.49	51.88	70.82

### 5.3 Discriminant Validity

To assess the discriminant validity of the CARS2, the total scores of individuals with autism, ID, and typical development (TD) were compared. The results indicated that the CARS2 effectively differentiates between the three groups.

Table 4 shows the mean, SDs, median, and range scores for the three groups who were administered the CARS2‐ST. The scores of individuals assessed as having autism were significantly higher than those with intellectually disabled (Mann–Whitney *U* = 2011. 50) and significantly higher than typically developing persons (Mann–Whitney *U* = 42.50). There was a significant difference in CARS2 scores between those with ID and typically developing groups (Mann–Whitney *U* = 83, *p* < 0.001) but the effect size is moderate, based on the test statistic value between individuals with autism and ID.

**TABLE 4:** The mean, SDs, median, and range scores of CARS2‐ST for the three developmental groups.

Development trajectory	*N*	Mean	Std. deviation	Median	Minimum	Maximum	Mann–Whitney *U*	*p*
Autism	166	39.27	9.172	39.50	15.50	84.50	Autism/ID 2011. 50	0.001
Intellectually disabled	97	25.45	7.795	24.00	16.00	50.00	ID/TPD 83.00	0.001
Typically developing	50	15.70	0.795	15.50	15.00	19.00	TPD/Autism 42.50	0.001

A similar analysis was undertaken of scores on the CARS2‐HF, and Table 5 shows that individuals with high‐functioning autism had significantly higher scores than typically developing individuals (Mann–Whitney *U* = 7080, *p* < 0.001).

**TABLE 5:** The mean, SDs, median, and range scores of CARS2‐HF for the two developmental groups.

Development trajectory	*N*	Mean	Std. deviation	Median	Minimum	Maximum	Mann–Whitney *U* Test	*p*
Autism	178	31.65	6.863	31.15	15.00	52.50	7080.0	0.000
Typically developing	40	15.62	0.657	15.50	15.00	17.50		

The discrimination validity was further checked through ROC analyses with both autism and the two non‐autism groups using the original cut‐off scores of < 30 for under 13 years individuals and < 27.5 for over 13 years old for CARS2‐ST and < 28 for CARS2‐HF. Table 6 summarizes the results of the ROC analyses that were undertaken considering the original cut‐off scores to differentiate individuals with autism and from the other two groups.

**TABLE 6:** Outcomes from the ROC analyses based on the original cut‐off scores.

	Cut‐off	Area	SE	Sig	Sensitivity	Specificity	Frequencies and percentage of the participants who had CARS score above the cut‐off.
CARS2‐ST < 13 years	30	0.932	0.017	0.000	0.886	0.10	Autism = 86 (59%) ID = 6 (7%) NTD = 0
CARS2‐ST ≥ 13 years	27.5	0.914	0.018	0.000	0.904	0.53	Autism = 13 (68%) ID = 3 (30%) NTD = 0
CARS2‐HF	28	0.994	0.006	0.000	0.940	0.280	Autism = 117 (66%) NTD = 0

Due to the low sensitivity of the original CARS2‐ST cut‐off scores particularly in younger individuals, new cut‐offs were determined using the Youden index method. As Table 7 shows the new cut‐off scores improved sensitivity for both CARS2‐ST and CARS2‐HF versions, meaning more individuals with autism are correctly identified from TD children. However, this comes at the cost of slightly decreased specificity, meaning more individuals without autism might be incorrectly classified. The recommended cut‐off for CARS2‐HF maintains high sensitivity and specificity, suggesting it might be a more balanced option for the use of CARS2 in Iran.

**TABLE 7:** Outcomes from the ROC analyses based on the recommended cut‐off scores.

	Cut‐off	Area	SE	Sig	Sensitivity	Specificity	Frequencies and percentage of the participants who had CARS score above the cut‐off.
CARS2‐ST < 13 years	27	0.921	0.000	0.000	0.964	0.503	Autism = 124 (84%) ID = 11 (13%) NTD = 0
CARS2‐ST ≥ 13 years	25	1.00	0.000	0.000	0.961	0.503	Autism = 15 (79%) ID = 4 (40%) NTD = 0
CARS2‐HF	22	1.00	0.000	0.000	0.994	0.550	Autism = 170 (95%) NTD = 0

Finally, as a check, the analyses were re‐run to determine whether the person's sex had an effect but no significant differences were found. Hence, individuals' sex did not appear to influence the tool's reliability, validity, or diagnostic accuracy using both the original and proposed cut‐off scores.

### 5.4 Qualitative Information

Parents in each ST and HF group were asked to describe in their own words their child's developmental difficulties and aby diagnosis they had been given. A summary of their comments for each group is presented in Tables 8 and 9.

**TABLE 8:** Parental descriptions of their child's development difficulties in the CARS2‐ST group (*N* = 313).

Developmental trajectory group	Parental description—*N* (percentage)
Autism (*n* = 166)	– Autism/ASD (85, 52%) – Mild, moderate or severe forms of Autism (17, 10%) – Communication disorders (11, 7%) – Autism and one accompanying diagnosis such as ADHD, learning problem (11, 7%) – Speech Disorder, speech problem (10, 6%) – Autism‐like features (9, 5%) – Abnormal (7, 4%) – Not so sure, not discovered, not known (5, 3%) – Behavior Problem (4, 2%) – Mental Problem (4, 2%) – Intellectual Disabilities (3, 1.5%) – Baby digital syndrome (1, 0.5%)
ID (*n* = 97)	– Intellectual disabilities (32, 33%) – Developmental disorders (27, 28%) – Mental delay/Problem (18, 18.5%) – Autism (10, 10%) – Attention Deficit Hyperactivity Disorders “ADHD” (5, 5.5%) – Learning Disability (5, 5.5%)
TD (*n* = 50)	– Normal Range, normal, ordinary, having no problem (49, 98%) – Slightly lack of attention (1, 2%)

**TABLE 9:** Parental descriptions of their child's development difficulties in the CARS2‐HF group (*N* = 218).

Developmental trajectory group	Prenatal description—*N* (Percentage)
Autism (*n* = 178)	Mild Autism (65, 36.5%) Moderate Autism (52, 29%) Autism along with another diagnosis (20, 11%) Speech delay/severe speech and communication delay (14, 8%) Unknown form of Autism (8, 5%)ADHD (5, 3%) Mental problem (4, 2%) Abnormal (3, 1.5%) Not very sure but something is wrong (3, 1.5%) Severe autism (3, 1.5%) Strange mood (1, 0.5%)
TD (*n* = 40)	At the normal range/completely typical/No problem/normal/ordinary/regular/typical (40, 100%)

The majority of the parents of individuals with autism in the ST and HF group used the term “autism” along with words to determine the level of severity of the symptoms. However, some parents used broad terms such as “developmental delay.” There were parents who used terms such as “abnormal,” “different,” “strange mood,” or “mental problem.” Nevertheless, the way parents describe their child's developmental condition can reveal their understanding of specific symptoms associated with different conditions. For instance, frequent use of terms such as “delayed speech” or “social awkwardness” might indicate awareness of autism spectrum symptoms.

### 5.5 CARS2‐QPC

The CARS2‐QPC is an unscored form completed by the parent or caregiver of the individual being assessed. Its purpose is to give the clinician more information on which to base CARS2‐ST or CARS2‐HF ratings and consists of seven sections that cover an individual's early development; social, emotional, and communication skills; repetitive behaviors; play and routines; and unusual sensory interests. The CARS2‐QPC serves as the framework for a follow‐up interview, during which the clinician can clarify and interpret the responses provided by the parent or caregiver.

For this study, 30 parents of persons with autism (15 from the CARS2‐ST and 15 from CARS2‐HF) were randomly selected (Greenbook, 2020). Parents were questioned by assessors based on the feedback they provided to QPC items, mainly in relation to Sections 1 (communication) and 7 (Special talents) while sections 4 (using senses) and 6 (play) received the least feedback.

Overall, parents highlighted the unique challenges faced by individuals with autism, emphasizing the importance of understanding their communication, emotional, sensory, and social needs when devising intervention plans.

### 5.6 Assessors' Views on CARS2

The study involved 20 administrators who when asked about the applicability of the CARS2 scale with these Iranian samples, all scored over 80 on a scale of 0–100. They did not come across any culturally sensitive items during the test administrations. However, they found the ST and HF forms to be the most applicable rather than CARS2‐QPC. Moreover, they thought that other scales with which they were familiar, such as the third version of Psychoeducational Profile (PEP3) should be used along with CARS2 because together they would provide a broader assessment of a child's cognitive and adaptive functioning, which can help identify additional areas of concern or strengths that may be relevant to an autism diagnosis. Particularly, it would help to differentiate autism from other developmental disorders that may present with similar symptoms, such as ID.

They thought that the QPC section was the most challenging as parental sensitivity regarding an autism diagnosis for their children could impact the professional's final diagnostic judgment. One evaluator said that “the QPC section often requires parents to reflect on their child's behavior in depth, which can be emotionally taxing and time‐consuming for them.” Another evaluator said that “Parental anxiety or denial about a possible autism diagnosis and its social consequences can sometimes influence their reporting of symptoms.” She concluded her justification by saying that “While involving parents in the diagnostic process is important for building trust, it's essential to be aware of the potential biases that may arise.”

## 6 Discussion

The purpose of the current study was to evaluate the appropriateness of using CARS2 in Iran by investigating the psychometric properties with an Iranian sample of individuals with autism and other types of developmental trajectories. CARS2 was broadly reliable in terms of internal consistency and test–retest reliability but less so for inter‐rater reliability a similar finding was reported with the Korean version of the scale (Lee, Yoon, and Shin 2023). However, it is important to note that the very high test–retest reliability by the same rater could have been inflated by potential biases such as practice effects, rater memory, and assessors' prior familiarity with participants. Encouragingly, the discriminant validity based on the total scores of the two versions can be used to differentiate between individuals with autism and typically developing children but less between those with ID. Nonetheless, the findings suggest that some adaptations are required when using CARS2 in Iran and possibly other cultures.

### 6.1 CARS2 Factor Structures

Factor analysis of the CARS2‐ST (DiLalla and Rogers 1994; Magyar and Pandolfi 2007) and CARS2 (Schopler et al. 2010; Lee, Yoon, and Shin 2023) has yielded diverse structural models across different studies, although they generally align with the core symptoms of autism as defined by the DSM‐5 (APA 2022). In particular, the social interaction and communication factor reflect the social impairments characteristic of autism, the emotional responses factor captures the emotional dysregulation often observed in individuals with autism, and the responsiveness factor assesses the restricted interests and repetitive behaviors associated with autism. Also, the results of the present study are close to the cumulative variance in the study conducted by Schopler et al. (2010) but here the principal components analysis of the CARS2‐ST items revealed one factor that accounted for 55% of the variance rather than the three factors identified in the original version (Schopler et al. 2010). A varimax rotation was used to replicate the three‐factor structure on which the items were based. This too, identified one main factor that accounted for 38% of the variance, with high loadings on the two domains of communication and social interactions that are indicative of autism and which are arguably more pertinent to an Iranian culture which emphases the style of communication expected of children (Habibi et al. 2017). We venture to suggest that total scores on the scale, rather than the subscale scores, should be used to make decisions about a diagnosis of autism, but cultural nuances and further data on the use of CARS2 with Iranian populations would help to refine the wording of items on this scale.

The three‐factor structure extracted from the CARS2‐HF data aligns with the original factor structure identified by Schopler et al. (2010) and accounted for a total of 62% of the variance. Factor 1 (27% of variance) reflected thinking/cognitive integration skills, Factor 2 (24%) emphasized social–emotional understanding, and Factor 3 (19%): focused on adaptation to change/restricted interests.

Each factor contributes a significant portion of the explained variance, which supports the validity of the three‐factor structure. Hence, CARS2‐HF could be recommended as a diagnostic tool for high‐functioning autism in Iran given its good reliability, validity, and discrimination ability. This is noteworthy given the lack of appropriate instruments for early detection and diagnosis of this group of individuals in Iran and regionally and many do not gain access to intervention services.

### 6.2 Cut‐Off Scores

In both versions of CARS2, there was a clear distinction between persons with autism and those with typical development. Moreover, the new cut‐off scores demonstrate a significant improvement in identifying children with ASD, particularly in younger age groups. This is a positive development as early diagnosis can lead to better outcomes. While there might be a slight increase in false positives, the overall benefits of improved sensitivity and reduced false negatives outweigh this concern.

However, the sensitivity around cut‐off scores was higher than specificity rates in CARS2‐ST in particular. Thus, individuals with ID might also be considered to have autism. This finding has already been reported for CARS2 with the Korean sample (Ji, Park, Yoon, and Hong 2023) and in the ADI‐R application for the Iranian sample (Samadi, McConkey, and Mahmoodizadeh 2021). While the exact source of this discrepancy remains unclear, it is possible that factors such as cultural differences in symptom presentation, variations in diagnostic criteria, or the absence of a “gold standard” diagnostic process may contribute to a failure in solely identifying individuals as autistic. The possibility of autism being a co‐morbidity with ID is another consideration. Gathering additional information, such as medical history, developmental milestones, and behavioral observations, to supplement the CARS2 data will further help in making a sound assessment on which suitable interventions can be devised. In general though, CARS2 holds promise for use in Iran given its reliability, construct validity, and discriminatory potentially using the adapted cut‐offs. Its suitability is further confirmed by those studies that have evaluated its use with Korean (Lee, Yoon, and Shin 2023) and Arabic versions of CARS (Alotaibi and Alotaibi 2021; Akoury‐Dirani, Alameddine, and Salamoun 2013) which also reported high internal consistency, inter‐rater reliability, and test–retest reliability. Thus, CARS2 could be recommended for use in cross‐cultural studies of prevalence and interventions to support persons with autism and family carers.

### 6.3 Family Caregivers' Perceptions of Autism

CARS2‐QPC has been rarely featured in past studies. However, the qualitative data from Iranian parents of individuals with autism suggest that the CARS2‐QPC can be a useful tool for collecting autism‐related information from parents and caregivers in Iran, although the assessors warned of some reservations that would warrant further observations of the child and discussions with parents. Similar findings were reported in another qualitative study by Hartl et al. (2022) for European caregivers.

By triangulating the qualitative and quantitative data, a more comprehensive understanding of the applicability of the CARS2‐ST and ‐HF for individuals with autism in Iran can be obtained. The qualitative data provide insights into the experiences of Iranian families with children with autism, while the quantitative data provide evidence for the validity of the scale in assessing the core symptoms of autism. This triangulation should help clinicians to make more informed decisions about the diagnosis and treatment of autism in Iran.

Nonetheless, the qualitative data provided by using CARS2‐QPC confirmed the importance of cultural considerations that should be taken into account when using the CARS2 in Iran. For example, parents primarily expressed concern over their children's speech delays, difficulties with communication, and social interaction than parents in other countries (Samadi and McConkey 2015; Samadi, McConkey, and Kelly 2012). This is likely due to cultural norms in Iran that emphasize politeness and indirect communication. Therefore, clinicians need to be aware of these cultural differences when interpreting the results of the CARS2‐QPC. Also, parental stress, anxiety, and social stigma surrounding autism may also influence symptom reporting (Poorkhorshidi et al. 2023). While parental involvement is vital in diagnostics, awareness of potential biases is important, and using standardized questioning along with additional scales focused on parents’ personal needs, such as emotional wellbeing, would help to triangulate information from multiple sources and guide the supports provided to help the person and family carers (Samadi, McConkey, and Bunting 2014).

A potential limitation of this study was the prior identification of autism among assessors and parents. This knowledge could have influenced their responses, potentially inflating the reliability data and skewing the factor structure. Additionally, the test–retest reliability was assessed by raters familiar with the child thereby limiting the gathering of new insights. Furthermore, due to practical constraints, it was not feasible to update the individual's assessments from the time of initial diagnosis, so previously ADI‐R and GARS2 data had to be used. While the CARS2 remains a valuable tool for assessing developmental difficulties, future research could benefit from recruiting a sample of children and parents who are unaware of their child's autism diagnosis and conducting independent test–retest reliability assessments at the time of initial diagnosis. This would help to address the limitations identified in the current study and enhance the generalizability of the findings.

Additionally, longitudinal studies can provide valuable insights into the long‐term validity and clinical utility of the adjusted CARS2 cut‐offs. By tracking the development of children identified using these cut‐offs over time, researchers can assess their outcomes and compare them to children diagnosed with other methods.

## 7 Conclusions

The factor analysis of CARS2‐HF data from individuals with high‐functioning autism provides evidence for the validity of the scale in assessing the core symptoms of autism. The three‐factor structure aligns with the DSM‐5 diagnostic criteria and can guide both diagnosis and intervention. For those with more severe symptoms of autism, the total scale score on CARS2‐ST with the revised cut‐offs could reliably and validly identify autism, although additional checks should be made to clarify the presence of intellectual impairments.

The qualitative data presented in this study provided further evidence for the validity and applicability of the CARS2‐ST and CARS2‐HF for individuals with autism in Iran. By triangulating these data sources with parental ratings and discussions, a more comprehensive understanding of the scale's utility was obtained. This information can help clinicians to better understand the challenges and parental expectations regarding the strengths of children with autism in Iran, and they can also help to improve the accuracy of diagnosis and early intervention services in the country.

## Conflicts of Interest

The authors declare no conflicts of interest.

## Data Availability

The data that support the findings of this study are available on request from the corresponding author. The data are not publicly available due to privacy or ethical restrictions.
